# Long-acting somatostatin analogs and well differentiated neuroendocrine tumors: a 20-year-old story

**DOI:** 10.1007/s40618-023-02170-9

**Published:** 2023-08-15

**Authors:** A. Faggiano

**Affiliations:** Endocrinology Unit, Department of Clinical and Molecular Medicine, Sapienza University of Rome, Sant’Andrea Hospital, ENETS Center of Excellence, Via di Grottarossa 1038, 00189 Rome, Italy

**Keywords:** Long-acting somatostatin analogs, Octreotide, Lanreotide, Neuroendocrine tumors, Tumor response, High-dose, MEN1

## Abstract

**Purpose:**

The specific indications of somatostatin analogs (SSAs) in patients with neuroendocrine tumor (NET) emerged over the time. The objective of this review is to summarize and discuss the most relevant data concerning long-acting SSAs in NET.

**Methods:**

A narrative review was performed including publications focusing on therapy with the long-acting octreotide, lanreotide, and pasireotide in patients with NET.

**Results:**

Long-acting SSAs confirm to be a manageable and widely used tool in patients with NET. Both long-acting octreotide and lanreotide are safe as the short-acting formulations, while patient compliance and adherence is further improved. Together with some randomized phase-3 trials, many retrospective and prospective studies have been performed in the last 20 years revealing a variable but substantial impact on progression free survival, not only in gastroenteropancreatic but also in lung and unknown primary NETs. The most frequent tumor response to SSAs is stable disease, but an objective response can be observed, more frequently by using high-dose schedules and in MEN1-related pancreatic NETs. Low tumor burden, low tumor grade (G1 and low G2), good performance status and use as first-line therapy are the main predictive factors to SSAs in NET patients. Pasireotide has been evaluated in few studies. This compound remains a promising SSA and would deserve to be further evaluated as a potential additional indication in NET therapy.

**Conclusions:**

Long-acting SSAs are an effective and safe initial therapy of patients with well differentiated NET, allowing tumor growth as well as symptoms control for long-time in selected patients.

## Introduction

Somatostatin analogs (SSAs) have been initially developed as short-acting octapeptide analogs of the native somatostatin and first employed for the treatment of neuroendocrine neoplasms (NENs) about 35 years ago. They were indicated in patients with carcinoid syndrome and other less frequent NEN-related endocrine syndromes, such as glucagonoma, VIPoma, etc [1–3]. The short-acting subcutaneous formulation of octreotide (OCT) is characterized by an 8-h half-life, administered two/three times a day, each single-dose ranging 0.05 to 0.5 mg [3, 4]. However, some studies reported experiences with high SSA daily dose, either with OCT or the other octapeptide lanreotide (LAN) [5–8].

Subsequently long-acting (LA) slow-release formulations have been developed. In particular, LAN slow-release and OCT LAR were developed for intramuscular injection at the dose of 30 mg every 14 days and 10–30 mg every 28 days, respectively [9–11]. Finally, the autogel LAN formulation was developed for deep subcutaneous injection at the dose of 60–120 mg every 14–28 days [12]. These compounds rapidly replaced the short-acting formulations being more manageable tool for clinical practice [4]. Pharmacokinetic studies of LA SSAs highlighted that the mean time to reach maximum concentration (t_max_) was 22 days for a dose of 20 mg OCT, 12.6 days for a dose of 60 mg OCT, while t_max_ was 2.4 days and 1.1 days for LAN 90 and 120 mg, respectively [13, 14]. Despite the different t_max_, the steady-state concentration was satisfying for both agents, resulting in a standard dose of 10–30 mg every 28 days for OCT LAR, 30–60 mg every 14–28 days for slow-release LAN, 60–120 mg every 14–28 days for LAN autogel. These formulations dramatically changed the patient’s perspective, improving the patient’s compliance to therapy and allowing long-time stable treatment. This finally resulted in an increase of studies investigating SSA effectiveness and safety in NEN patients. The pharmacokinetic findings above reported also suggest these drugs to be dose-dependent, supporting their use in high dose schedules, at least in NEN patients refractory to standard doses. This approach is now accepted in the last NEN guidelines and will be described in this review in a dedicated paragraph.

LA SSAs represent nowadays the first-line treatments of low-grade advanced well differentiated NENs, the so called neuroendocrine tumors (NETs). Both LA OCT and LAN demonstrated to improve the rate of progression in randomized placebo-controlled trials [15–17]. Even before these trials, LA SSAs have been used in clinical practice, first in patients with NEN-related endocrine syndromes to control hormone hypersecretion and related symptoms, then also in non-functioning NETs, mostly G1-G2, as antiproliferative agents. These preliminary results have mostly been reported in several retrospective and few prospective studies (Table 1). More recently, other studies have been conducted to investigate the activity of LA SSAs in different conditions as NET with primary origin other than gastroenteropancreatic (GEP) or genetically determined NETs, as well as, also, focusing on above level schedules or new SSAs (as Pasireotide).

**Table 1:** Efficacy outcomes of non-randomized studies with long-acting somatostatin analogues in neuroendocrine tumors

First author, Journal, year	Type of study	Primary tumor site	N	Tumor grade	Tumor progression before SSA	Long-acting SSA	Progression free survival	Overall survival	Objective response rate
Panzuto F et al. Annals Oncolology 2006 [68]	Prospective	Pancreas: 18 Intestine: 11 Unknown: 2	31	G1: 14 ≥ G2: 13 NA: 4	81% NA in 19%	OCT 30 mg/28 d: 21 LAN 60 mg/28 d: 10	3-yr PFS: 74.1%	3-yr PFS: 75%	CR/PR: 0/0 SD: 45.2% PD: 54.8%
Khan MS et al. Alimentary Pharmacology & Therapeutics, 2011 [69]	Retrospective	Midgut: 69	69	G1: 48 G2: 17 G3: 4	NA	LAN 60 mg/28 d: 23 LAN 90 mg/28: 36 LAN 120 mg/28 d: 7	1-yr PFS: 94% 3-yr PFS: 70% 5-yr PFS: 45%	mOS: not reached	CR/PR: 0/0 SD: 54% PD: 46% (clinical and/or radiological)
Anthony L et al. Pancreas, 2011 [70]	Retrospective	Pancreas: 56 Stomach: 10 Duodenum/jejunum: 28 Ileum: 122 Appendix: 9 Colon: 27 Rectum: 5 Cecum: 26 Lung: 29 Other: 45	392	NA	NA	Evaluable for OCT 20 mg/28 d: 240 Evaluable for OCT 30 mg/28 d: 316	NA	NA	20 mg CR/PR: 2/6% SD: 57% PD: 21% 30 mg CR/PR: 1/8% SD: 57% PD: 25%
Bianchi A et al. Journal of Endocrinological investigation, 2011 [71]	Retrospective	Pancreas: 8 Stomach: 1 Duodenum: 1 Ileum: 4 Rectum: 1 Lung: 5 Unknown: 3	23	NA	100%	LAN 120 mg/m	mTTP: 28 mos (6–50 mos)	NA	GEP CR/PR: 0/0 SD: 73% PD: 23% Lung CR/PR: 0/40% SD: 40% PD: 20% Unknown CR/PR: 0/40% SD: 40% PD: 20%
Jann H et al. Neuroendocrinology, 2013 [39]	Retrospective	Pancreas: 43	43	G1: 8 G2 30 NA: 5	53% Unknown in 35%	OCT 30 mg/28 d: 19 OCT ≤ 20 mg/28 d: 16 NA: 8	mTTP: 13 mos (2–51)	mOS: 98mos (14–216)	CR/PR: 0/7% SD: 58% PD: 35%
Martín-Richard M et al. BMC Cancer, 2013 [41]	Prospective, single-arm phase-II study	Pancreas: 8 Stomach: 1 Small bowel: 10 Large bowel: 3 Lung: 4 Unknown: 4	30	G1: 13 G2: 8 NA: 9	100%	LAN 120 mg/28 d	mPFS: 12.9 mos (95% CI: 7.9–16.5 mos)	NA	CR/PR: 0/4% SD: 89% PD: 7%
Faggiano A et al. Oncotarget, 2015 [24]	Prospective / Retrospective	Pancreas: 49 Stomach: 8 Ileum: 15 Lung: 13 Thymus: 5 Other sites: 7 Unknown: 9	106	G1: 49 G2: 57	NA	OCT 30 mg/28 d: 71 LAN 120 mg/28 d: 35	GEP mPFS: 89 mos Lung*mPFS: 59 mos Unknown mPFS: 35 mos	NA	GEP CR/PR: 3/10% SD: 59% PD: 29% Lung* CR/PR: 0/6% SD: 61% PD: 33% Unknown CR/PR: 0/11% SD: 44% PD: 44%
Wang Y et al. Oncology Letters, 2017 [72]	Retrospective	Pancreas: 64 Rectum: 34 Stomach: 19 Duodenum: 15 Jejunum/ileum: 7 Appendix: 4	143	G1: 69 G2: 39 G3: 2 NEC:31 Manec:2	NA	OCT 20–40 mg/28 d	mTTP: 20.2 mos (95% CI, 13.9- 26)	mOS: not reached	CR/PR: 0/5.6% SD: 79.6% PD: 14.8%
Sullivan I et al. European Journal of Cancer, 2017 [73]	Retrospective	Lung: 61	61	TC: 20 AC: 41	100%	OCT 20 mg/28 d: 12 OCT 30 mg/28 d: 29 OCT 30 mg/14 d: 5 LAN 90 mg/28 d: 8 LAN 120 mg/28 d: 7	mPFS: 17.4 mos (95% CI: 8.7—26.0)	mOS 58.4 (95% CI: 44.2—102.7) mos	OR: NA SD: 77% PD: NA
Kang J et al. Investigational New Drugs, 2018 [37]	Retrospective	Pancreas 22 Rectum 10 Small bowel 7 Stomach 2	45	G1: 8 G2: 32 G3: 2 NA: 3	NA	LAN 90 mg/28 d: 8 LAN 120 mg/28 d: 37	mPFS: 16.4 mos (95% CI, 9.5–23.3)	mOS: not reached	CR/PR: 0/2.2% SD: 88.9% PD: 8.9%
Satapathy S et al. JCO Global Oncology, 2021 [74]	Retrospective	Pancreas: 14 Foregut: 8 Midgut: 8 Hindgut: 5 Unknown: 5	40	G1: 24 G2: 16	NA	OCT 30 mg/28 d	mPFS: 16 mos (95% CI, 13–18.9)	mOS: not reached	CR/PR: 3/12% SD: 52% PD: 33%

*NA* not available, *SSA* somatostatin analogues, *OCT* octreotide, *LAN* lanreotide, *PFS* progression-free survival, *CR*: complete response, *PR* partial response, *SD* stable disease, *PD*: progressive disease, *OS* overall survival, *TTP* time to progression, *GEP* gastroenteropancreatic, *thymic NETs included

This review aims to summarize data on efficacy and safety of LA SSAs in patients with NET but also to report some peculiar aspects which are not frequently focused on.

## Methods

This narrative review was performed for available prospective, retrospective and review articles, published up to April 2023 in PubMed. Data were extracted from the text and from the tables of the manuscript. The keyword search used included “somatostatin analogues and neuroendocrine tumors”, “somatostatin analogues and neuroendocrine neoplasms”, “somatostatin analogues and carcinoid syndrome”, “somatostatin analogues and inherited tumor syndromes”, “somatostatin analogues and MEN1”, “octreotide and neuroendocrine tumors”, “lanreotide and neuroendocrine neoplasms”, “octreotide and neuroendocrine neoplasms”, “lanreotide and neuroendocrine neoplasms”, “octreotide and inherited tumor syndromes”, “lanreotide and inherited tumor syndromes”, “octreotide and MEN1”, “lanreotide and MEN1”, “pasireotide and neuroendocrine tumors”, “pasireotide and neuroendocrine neoplasms”. The articles were selected on the basis of relevance of title and abstract in the topic.

## Results

### Efficacy and tolerability of octreotide and lanreotide

The main advantage associated with LA formulations has been to obtain a manageable tool for long-term therapy. Improved patient compliance has been reported in patients treated with LA formulations as compared to the short-acting ones, even if associated with a similar drug profile in terms of both effectiveness and safety [18–20].

SSA effectiveness in NET patients has been observed regardless from their anatomic origin, either to control hormonal symptoms or to exert antiproliferative activity [21–23]. The most robust data have been obtained in gastroenteropancreatic (GEP) NETs and in particular in ileal and pancreatic tumors which were investigated in randomized phase 3 trials [15, 16]. OCT LAR at the dose of 30 mg every 28 days demonstrated a significant improvement of the median time to progression vs placebo in patients with advanced midgut NET (14.3 vs 6 months, HR 0.34, *p* <0.0001) [15]. Notably, in this study 95.3% of the included patients had Ki-67 values up to 2%. LAN autogel at the dose of 120 mg every 28 days was associated with a significant improvement of the median PFS as compared to placebo in patients with advanced entero-pancreatic NET (not reached vs 18 months, HR 0.47, *p* <0.001) [16]. Among the 101 patients treated with LAN autogel, 69 cases (68%) had a Ki-67 of 0-2 % and 32 cases (32%) a Ki-67 up to 10%. A subsequent open-label extension study reported a median PFS of 38.5 months in LAN-LAN subgroup and a time to death or subsequent progression of 19 months in those treated with LAN at the time of progression [17]. If these studies definitively clarified the antiproliferative activity of LA OCT and LAN in low grade GEP NETs, on the other hand they demonstrated a satisfying safety profile with a low rate of treatment discontinuation, consistent with long-term treatment and good quality of life [15–17]. Many other non-randomized studies investigated the role of LA SSAs in real world, demonstrating variable results but confirming the activity of these compounds not only in GEP but also in lung NETs, as well as in metastatic tumors with unknown primary site, even though at a lower extent (Table 1). More in deep, non-randomized studies were highly variable for study design, patient population, SSA type and schedule. The median PFS ranged from 12.9 to 89 months mainly depending on tumor stage, grade and growth rate as well as previous treatments. The study reporting the highest PFS included mainly low-grade tumors (G1 and G2 NET in 79% of cases) and both localized and metastatic tumor stage [24]. The objective response rate varied at a lesser extent ranging from 0 to 15%, which was a partial response in the vast majority of cases. The most frequent type of response to SSA was stable disease, ranging from 45 to 89% (Table 1). Unfortunately, the development of resistance to SSA could occur and this issue should be taken into account in patients’ management. However, the molecular mechanisms involved in this complex phenomenon have not been elucidated, so far.

LA SSAs are safe as well documented not only in controlled trials but also in real-world studies [25]. Both treatment discontinuation and dose adjustment are unfrequently needed [26]. Gastrointestinal abnormalities are the most frequently reported side effects in this setting. They are generally mild to moderate and include abdominal cramps, steatorrhea and altered digestion [27]. One relevant but poorly investigated cause of gastrointestinal disorders as consequence of SSA therapy is the exocrine pancreatic insufficiency, which needs to be recognized and treated [28]. However, the most relevant side-effect to manage in patients treated with SSAs is cholelithiasis, ranging from biliary sludge to gallstones, which can be complicated by biliary colic, cholecystitis or cholangitis [29]. The rate of biliary disorders in patients with advanced GEP NET is not increased in LA as compared to short-acting SSAs [30]. This finding is also true for the whole spectrum of toxicity which does not differ between different SSA formulations [18]. The safety profile is not different between OCT and LAN. However, from a practical point of view, LAN was reported to be easier to manage than OCT and less frequently complicated by pain and technical problems, because of the autogel formulation with a comfortable device and a fast administration [31, 32].

### Predictive factors of response

Among the pathologic factors, somatostatin receptor (SSTR) expression is expected to play a central role to predict response to LA SSAs in NET patients. However, few evidences are available regarding the SSTR status in relation to SSA therapy. In a work by Kasajima et al. including 38 NEN patients, SSTR type 2 (SSTR2) expression evaluated through HER2-Score was associated with response to SSA treatment (*p* = 0.045) [33]. Volante et al. reported a positive correlation between immunohistochemical SSTR2A score 2/3, as defined by tumor cell membrane immunostaining, and response to treatment with octreotide LAR [34]. Conversely, a negative predictive role has been identified for poorly differentiated morphology [35], as well as higher tumor grade [36, 37]. The Ki-67 index, the main proliferative marker in NET, has been reported to be predictive for survival and progression outcomes in NET patients treated with SSA [24, 38]. Guidelines consider a threshold of 10% to suggest SSA therapy in advanced NETs [22], on the basis of Clarinet trial. A 10% cut-off is also supported by some retrospective studies [39, 40], while a 5% cut-off has been reported in other studies on GEP and lung NETs [24, 41].

With regards to clinical factors, a worse patient performance status [38], as well as the tumor growth rate at baseline have been identified as negative predictors of response to SSA [42]. In this context, the GETNE-TRASGU tool, elaborated by the Spanish Group of Neuroendocrine and Endocrine Tumors (GETNE) from the data of 535 patients with GEP-NENs receiving SSA treatment, has suggested that the presence of symptoms, the extent of liver involvement, the presence of bone and peritoneal metastases and the progression status, are all negative factors [43]. In the same work, also higher neutrophil-to-lymphocyte ratio and higher alkaline phosphatase levels have been identified as negative predictive factors for SSA treated patients [43]. Among the circulating biomarkers, conflicting data are available on chromogranin A (CgA). Few studies report the decrease of CgA after SSA therapy as a positive predictive factor [44, 45]. A post hoc analysis of the Phase 3 CLARINET study detected a correlation between CgA response to SSA and patients’ outcomes [45]. Interestingly, in this study a decrease of 5-hydroxyindoleacetic acid (5-HIAA) levels was associated to an improved PFS in SSA-treated patients. Forest plot analysis confirmed a correlation of 5‐HIAA reduction being favorable for SSA [45]. Finally, positive SSTR status on somatostatin receptor imaging (SRI) has failed to demonstrate to be predictive of response to SSA [46]. By a practical point of view, to check SRI positivity is recommended but not necessary for antiproliferative LA SSA therapy in NET [23]. On the other hand, it should be underlined that the optimal candidates for SSA therapy are well differentiated low-grade NETs, which are commonly SSTR2-positive and therefore expected to be SRI-positive.

### High-dose somatostatin analogs

Traditionally, a SSA dose increase is implemented to control refractory symptoms related to specific endocrine syndromes, such as carcinoid syndrome, glucagonoma etc. However, a role in tumor growth control has been recently recognized in the ENETS guidelines [47]. A non-randomized clinical trial dedicated to investigate above-label dose of LA SSAs has been only recently published in patients with pancreatic or midgut NETs treated with LAN 120 mg every two weeks, after progression with LAN at standard dose, reporting a median PFS of 8.3 months and 5.6 months, respectively [48]. Few phases 3 trials on digestive NETs with tumor progression or uncontrolled carcinoid syndrome on standard SSA therapy reported data on high-dose OCT as control arm. In particular, in the pasireotide trial OCT 40 mg monthly resulted in a mPFS of 6.8 months, while in the Netter-1 trial, OCT 60 mg monthly resulted in a mPFS of 8.4 months [49, 50]. No randomized trial focusing on high-dose SSAs has been performed. However, high-dose schedules with SSAs are widely used in clinical practice and studies on this topic dated from more than twenty years ago, firstly investigating short-acting agents then LA formulations. A conclusion shared by all studies is that adverse events are not increased by the increase of the dose. Regardless from SSA and type of schedule, high-dose treatments show similar safety profiles as standard treatments [23, 26, 51, 52]. Less definitive findings are reported about the efficacy outcomes of this approach. A recent metanalysis reported low rates of antiproliferative effect with high-dose after failure of SSAs at standard doses [53]. Most of data are obtained on low-grade GEP NETs. In this setting, median PFS ranges about 25–30 months in patients with radiologic or symptomatic progression under therapy with SSAs at standard doses. An objective tumor response was found in 4–14% of patients, according to different studies, patient and tumor characteristics, SSA schedule (Table 2). A large retrospective Italian Multicentric Study, performed on a homogeneous population of G1–G2 GEP NETs progressing on standard SSA treatments, highlighted median PFS 31 months, objective response 8.4%, stable disease 75.7% [54]. The early use in the therapeutic sequence was the only predictive factor of response to high-dose SSA. These findings are in line with a previous retrospective study comparing four different therapeutic sequences in GEP and lung G1-G2 NETs progressing on standard doses. The sequence with above-standard SSA in second-line was equally effective as the other sequences with everolimus, chemotherapy and PRRT respectively [55]. According to these studies as well as to the Clarinet forte trial [48], the increased dose density is the commonest schedule, although dose intensity is a further possibility to take in account. An ultra-high-dose LA OCT (160 mg every 28 days) has been tested in patients with progressive ileal NET in progression after standard doses, but this compound has not been further developed and commercialized [56]. In summary, high-doses seem to be a reliable and safe approach to late the progression and switch to more aggressive treatments. However, the real antiproliferative efficacy of this approach is still debated and needs to be better investigated in dedicated prospective trials. At now, dose frequency increase is the best schedule in clinical practice. The best candidate for high-dose SSA therapy reflects the same identikit of the candidate to standard SSA therapy. In particular, a Ki-67 cut-off of 5% is a recognized predictive factor both for standard and high doses [24, 57]. On the contrary, tumors with Ki-67 >5% but also high growth rate and tumor burden suggest different approach (PRRT, everolimus, chemotherapy) after failure of SSA at standard doses.

**Table 2:** Efficacy outcomes of non-randomized studies with high dose long-acting somatostatin analogues in neuroendocrine tumors

First author, Journal, year	Type of study	Primary tumor site	N. of pts on high dose	Tumor grade	Tumor progression before SSA	Long-acting somatostatin analogues	Progression free survival	Overall survival	Objective response rate
Anthony L et al. Pancreas, 2011 [70]	Retrospective	Pancreas: 56 Stomach: 10 Duodenum/jejunum: 28 Ileum: 122 Appendix: 9 Colon: 27 Rectum: 5 Cecum: 26 Lung: 29 Other: 45	120	NA	NA	OCT 40 mg/28 d: 78 OCT 60 mg/28 d: 42	NA	NA	40 mg CR/PR: 0/4% SD: 55% PD: 18% 60 mg CR/PR: 2/10% SD: 50% PD: 29%
Ferolla P, Faggiano A et al. Journal of Endocrinological Investigation, 2012 [75]	Prospective	Pancreas: 11 Stomach: 1 Duodenum: 1 Ileum: 8 Rectum: 1 Lung: 4 Thymus: 2 Unknown: 1	28	NA	100%	OCT 30 mg/21 d	30 mos (95% CI, 24.7–35.3)	NA	CR/PR: 0/7.2% SD: 92.8%
Faggiano A et al. Oncotarget, 2015 [24]	Prospective / Retrospective	GEP/lung/thymus	14	G1/G2	64.3%	OCT 30 mg/21 d: 4 OCT 30 mg/14 d: 2 LAN 120 mg/21 d: 4 LAN 120 mg/14 d: 4	NA	NA	OR: 0/14.3% SD: 71.4% PD: 14.3%
Lamberti G et al. Journal of Clinical Endocrinology & Metabolism, 2020 (54)	Retrospective	Pancreas: 43 Gastrointestinal tract: 97	140	G1: 75 G2: 63 NA: 2	100%	LAN 180 mg/28 d or OCT 60 mg/28 d: 7 LAN 120 mg/14 or/21 d or OCT 30 mg/14 or /21 d: 133	31 mos (95% CI, 19.3–42.6)	NA	CR/PR: 0/8.6% SD: 75.7% PD: 15.7%
Diamantopoulos LN et al. Neuroendocrinology 2021 [57]	Retrospective	Pancreas: 8 Small intestine: 85 Colorectal: 12	105	G1: 48 G2: 39 G3: 1 NA: 17	35%	OCT 30 mg/21 d: 60 LAN 120 mg/21 d: 45	mPFS: 25 mos (95% CI:17–33)	mOS: 136.9 mos (95% CI: 66–207)	SD: 51% PD: 49% (evaluated on 37 patients)
Pavel M et al. Eur J Cancer 2021. [48]	Prospective	Pancreas: 48 Small intestine: 51	99	G1: 41 G2: 58	100%	LAN 120 mg/14	Pancreatic mPFS: 5.6 mos (95% CI, 5.5–8.3) Small intastinal mPFS: 8.3 mos (95% CI, 5.6–11.1)	NA	Pancreatic CR/PR: 0/0% SD: 66.7% PD: 31.3% Small intestine CR/PR: 0/3.9% SD: 68.6% PD: 23.5% (evaluated on 50 patients)

*NA* not available, *SSA* somatostatin analogues, *OCT* octreotide, *LAN* lanreotide, *PFS* progression-free survival, *CR* complete response, *PR* partial response, *SD* stable disease, *PD* progressive disease, *OS* overall survival, *GEP* gastroenteropancreatic

### NET in patients with inherited tumor syndromes

No specific indications are available for therapy with LA SSAs in NETs associated to inherited syndromes. Up to 10% of NENs can occur in the context of hereditary syndromes, such as multiple endocrine neoplasia type 1 (MEN1), multiple endocrine neoplasia type 2 (MEN2), multiple endocrine neoplasia type 4 (MEN4), Von Hippel–Lindau (VHL), neurofibromatosis type 1 (NF1), and tuberous sclerosis [58], the most frequent localization being the pancreas [58, 59]. MEN1 pancreatic NETs are the most frequent genetic NENs. These represent autosomal dominant inherited tumors, characterized by multifocality, very low grade and high SSTR expression. MEN1-related pancreatic NETs appear as optimal candidate to SSA therapy even at localized non-metastatic stage. According to the available evidences the objective tumor response was obtained with LA SSA in 10-33% of MEN-1-related pancreatic and duodenal NET patients (Table 3). A recent review systematically analyzed the efficacy and safety of SSA treatment in patients with MEN1-related pancreatic NETs [60]. Overall, 20 studies comprehensives of 105 MEN1 patients were included. Tumor response to SSAs was higher in MEN1-related NETs as compared to the sporadic counterpart. Specifically, stable disease (SD) was found in 75.6% of cases, while an objective response occurred in 12.7%. In particular, 8.9% of patients showed a partial response (PR), 3.8% a complete response (CR). No significant differences were observed in terms of efficacy between OCT and LAN. The only MEN1-dedicated prospective study with SSAs at standard dose evaluated LAN 120 mg monthly vs active surveillance. LAN was able to induce an objective tumor response in 17.4% and stable disease in 65.2% of patients with MEN1 pancreatic NETs <2 cm, while the median PFS was not reached vs 40 months in the group of patients without treatment [61].

**Table 3:** Objective tumor response of long-acting somatostatin analogues in MEN1-related neuroendocrine tumors

First author, journal, year	Type of study	N. of patients	Tumor site	Type of long-acting SSA	Objective tumor response
Shojamanesh H al Cancer, 2002 [76]	Prospective	3	Pancreas and/or duodenum	Octreotide 20 mg/28 days	CR/PR: 0/33% SD: 33% PD: 33%
Ramundo V et al. Clinical Endocrinology, 2014 [77]	Retrospective	20	Pancreas and duodenum	Octreotide 30 mg /28 days	CR/PR: 0/10% SD: 80% PD: 10%
Cioppi F et al. Clinical Cases in Mineral and Bone Metabolism, 2017 [78]	Prospective	8	Pancreas	Octreotide 10 mg/28 days	CR/PR: 12.5%/0 SD: 87.5%
Oleinikov K et al. Endocrine, 2020 [79]	Retrospective	12	Pancreas	NA	CR/PR: 0/0 SD: 92% PD: 8%
Faggiano A et al. Journal of Clinical Endocrinology & Metabolism, 2020 [61]	Prospective	23	Pancreas	Lanreotide 120 mg /28 days	CR/PR: 17.4% SD: 65.2% PD: 17.4%

*NA*: not available, *MEN1*: multiple endocrine neoplasia type 1, *SSA*: somatostatin analogue, *CR*: complete response, *PR*: partial response, *SD*: stable disease, *PD*: progressive disease

Different reasons have been proposed to explain the better therapeutic response to SSAs observed in MEN1 than in sporadic pancreatic NETs. First, these patients showed a high rate of early stage and localized disease. Second, they are well differentiated low-grade tumors, with Ki-67 index usually <5% and high SSTR2 expression. Third, functioning NETs are more frequent in MEN1. All these characteristics make MEN1-related pancreatic NETs highly sensitive to SSA treatment [60].

Finally, in NEN associated with MEN2, NF1, VHL, and tuberous sclerosis data on the efficacy of SSAs are scarce and derive generally from case reports [62, 63]. In absence of specific indications, NETs associated with these syndromes can be treated in analogy with the MEN1-related NETs.

### Pasireotide

**Fig. 1:**
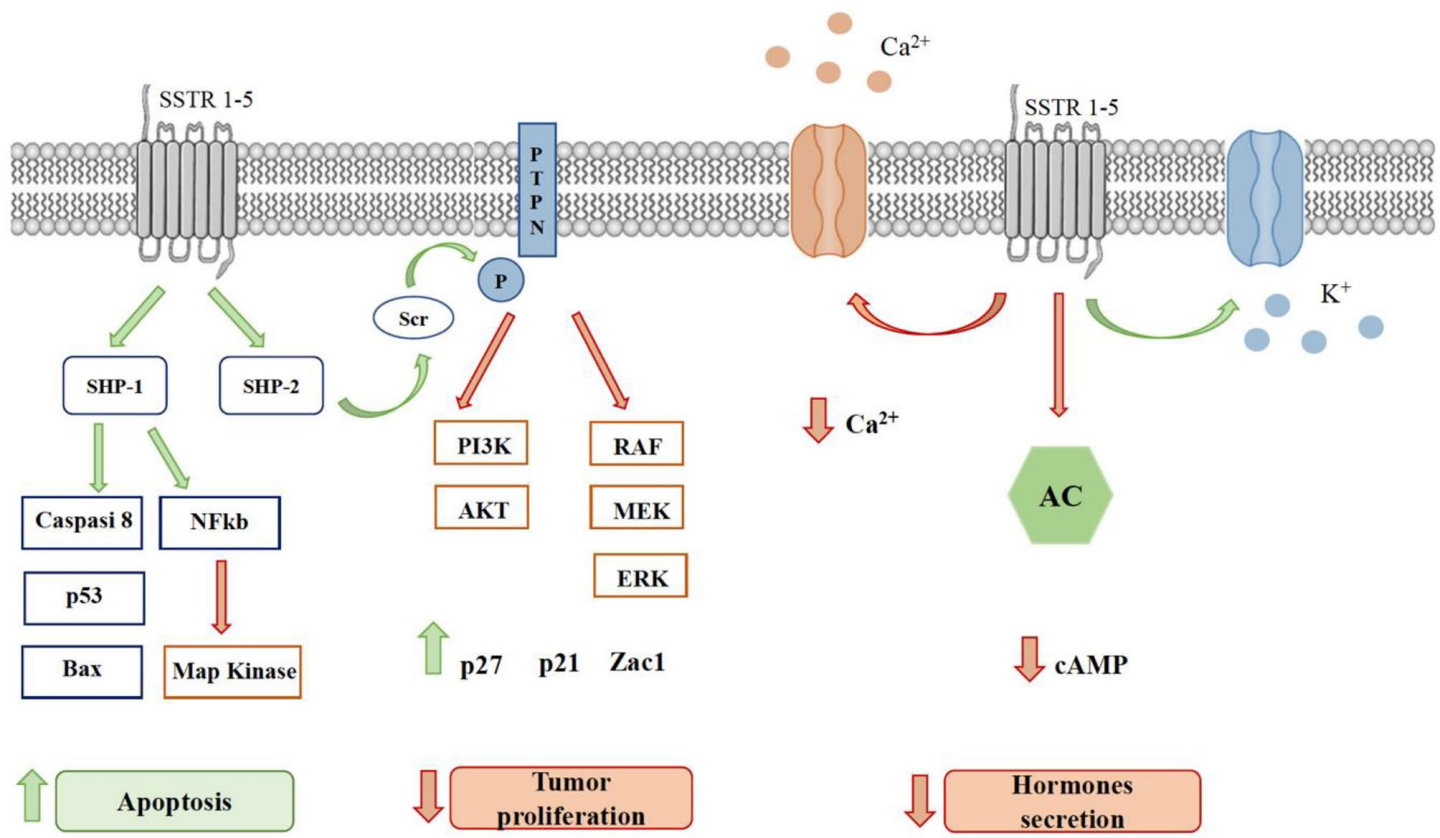
Intracellular signal pathway of somatostatin receptors. SSTR: somatostatin receptor; PTPN: protein tyrosine phosphatase; PI3K: phosphoinositide-3-kinase; AKT: Protein kinase B; AC: adenylyl cyclase

Pasireotide is a second generation SSA which has binding affinity for SSTR1, 2, 3, and 5. Pasireotide is available in a short-acting formulation for subcutaneous (SC) injection, which is administered twice daily, and a LA formulation for intramuscular (IM) injection, which is given every 28 days. Both formulations exhibit similar pharmacokinetic and pharmacodynamic properties and have a comparable safety profile [64, 65]. LA pasireotide is a promising therapy for patients with NET, especially those refractory or resistant to other SSAs [65] (Figure 1).

Yao et al. in a phase I study, demonstrated that the maximum tolerated dose for pasireotide in patients with advanced NETs is 120 mg, at which dose bradycardia events reached 31% compared to 0% at the 80 mg dose. The same authors highlighted encouraging effects of pasireotide on PFS and disease control, as well as on reduction of tumor markers (CgA, NSE, IGF-1). The pharmacokinetic profile of LA pasireotide in patients with NET revealed that this compound maintains steady plasma concentrations over the 28-day dosing interval with no evidence of accumulation in the body [66]. Furthermore, pasireotide was generally well-tolerated despite a high rate of adverse effects. The most common adverse events of any grade observed were hyperglycemia, fatigue, but also diarrhea and nausea [66]. Focusing specifically on hyperglycemia, in this study this side effects accounted for 79.3% of cases, with grade 3–4 in 10.3% of patients (a grade 3–4 was reported in 15.4% of cases with LA pasireotide 80 mg and in 6.3% of patients treated with LA pasireotide 120 mg). A good profile of safety and tolerability of LA pasireotide at a maximum dose of 60 mg has been then reported in patients with GEP-NET [49]. In a phase II prospective clinical trial, LA pasireotide 60 mg was evaluated in 29 NET patients as first-line systemic therapy. In this study 13 patients had a low-grade NET and 16 an intermediate grade tumor. The median PFS was 11 months (95% CI 7.6–16 months) [67]. A rate of 4% of patients experienced a partial response, 60% a stable disease and 36% progressive disease. In line with previous studies, adverse effects were mild to moderate, with high prevalence of hyperglycemia (65%), followed by diarrhea (14%) [67]. In a phase III study, two different SSA therapies, LA pasireotide (60 mg) and OCT (40 mg), were compared in patients with metastatic NET and carcinoid symptoms refractory to first-generation SSAs. The included patients had a well differentiated tumor in 77% of cases in the LA pasireotide arm and in 84% of cases enrolled in OCT arm. The study was halted at an interim analysis following a data monitoring committee recommendation due to a low predictive probability of showing superiority of pasireotide over OCT for carcinoid symptoms control. However, pasireotide group had a longer median PFS as compared to OCT (11.8 versus 6.8 months, *p*=0.045), suggesting a better antiproliferative activity of pasireotide [49].

## Conclusions

LA OCT and LAN are an effective and safe tool for long-term therapy of patients with NET. In the last twenty years many evidences have been reported not only from selected patients included in randomized trials but also from real-world patients included in different types of retrospective and prospective studies. These compounds impact significantly on progression free survival and disease control rate, despite the variable entity of the response observed, according to study design, SSA schedule, study population and tumor characteristics. When used in patients with good performance status, with low-tumor burden and low-grade NETs, even better if with Ki-67 index ≤5%, and as first-line therapy, SSAs ensure prolonged progression free survival and a low but not negligible objective response rate, together with good control of endocrine symptoms and life quality, while severe adverse events and treatment discontinuation are rarely reported. In the same setting, SSAs administered at high doses, more easily with a dose-frequency increase, seem to be an effective second-line therapy in NETs progressing on standard doses. In the peculiar setting of MEN1, SSAs seem to be even more effective than in sporadic NETs. Finally, pasireotide remains a promising SSA and would deserve to be further evaluated as a potential additional indication in NET therapy.

## Declarations

### Human and animals rights

The study does not involve human participants and/or animals.
